# Synergistic Antioxidant Effects of Cysteine Derivative and Sm-Cluster for Food Applications

**DOI:** 10.3390/antiox13080910

**Published:** 2024-07-28

**Authors:** Lingxia Chen, Lijun Wang, Lifu Ma, Chao Wang, Xinshu Qin, Minlong Wang, Xiaohe Zhang, Ruoyan Yang, Bing Fang, Jie An

**Affiliations:** 1 Department of Nutrition and Health, China Agricultural University, Beijing 100083, China; lxchen@cau.edu.cn (L.C.); lijunwang@cau.edu.cn (L.W.); b20233311213@cau.edu.cn (C.W.); b20233311203@cau.edu.cn (X.Q.); mlwang@cau.edu.cn (M.W.); b20223311102@cau.edu.cn (X.Z.); 2019310060114@cau.edu.cn (R.Y.); 2 Tianjin Rianlon Corporation Research Institute Analytic Center, Tianjin 300457, China; malifu@rianlon.com

**Keywords:** oxidation, antioxidant, cysteine derivative, Sm-cluster, thiol, DPPH

## Abstract

The incorporation of antioxidants in food products is essential to prevent or delay deterioration, thereby addressing food spoilage. Thiol compounds, recognized for their natural antioxidant properties, are widely used in various foods; however, their antioxidant capacity is often limited. This study investigates the potential enhancement of thiol antioxidant capacity through the addition of a soluble, low-toxic inorganic Sm-cluster. Our findings demonstrate that the Sm-cluster significantly bolsters the antioxidant efficacy of thiol compounds. We explored, for the first time, the in vitro antioxidant activities of an Sm-oxo/hydroxy cluster combined with a cysteine derivative for potential food applications. The composition exhibited a robust inhibition of aromatic aldehyde flavor compound oxidation and displayed strong, dose-dependent DPPH (2,2-diphenyl-1-picrylhydrazine) radical scavenging activity. Notably, the antioxidant activity of the Sm-cluster/cysteine derivative was further enhanced under strong visible light conditions, which typically increased the likelihood of oxidation. These results suggest that the combination of inorganic cluster and thiol compounds presents a promising natural alternative to traditional antioxidants in the food industry.

## 1. Introduction

Food spoilage has long been a major concern, particularly in countries with relatively warm climates [1]. The process of food spoilage is closely linked to oxidative reactions and the decomposition of oxidative products [2]. To prevent or delay food spoilage, antioxidants are widely used as additives in food processing [3]. They play a crucial role in the food industry and are one of the most common methods of food preservation. Antioxidants used in the food industry can be classified into natural antioxidants and synthetic organic antioxidants.

Biological thiols, or biologically derived thiols, are among the most important natural antioxidants [4]. Examples include glutathione, cysteine, N-acetylcysteine, homocysteine, and γ-glutamylcysteine [5,6]. However, despite their potential, thiol antioxidants often suffer from limited antioxidant potency. Although synthetic antioxidants, such as butylated hydroxytoluene and tert-butylhydroquinone [7], are more effective compared to natural antioxidants, they have been shown to pose potential health hazards, including toxicological and carcinogenic effects [8].

In recent years, many less toxic inorganic nanoparticles with antioxidant properties (average size < 100 nanometers) have been discovered to exhibit excellent antioxidant performance [9,10]. Examples include CeO_3_, TiO_2_, and Fe_3_O_4_ [11,12,13]. In addition to their standalone use, inorganic nanoparticles can enhance the antioxidant activity of organic substances through synergistic effects [14,15]. Nanoparticles have been incorporated into various biodegradable polymer matrices to create enhanced nanocomposites with high performance and improved properties [16,17,18]. For instance, samarium (Sm) and its oxides are widely used in many products due to their low toxicity [19]. Although inorganic nanoparticles are generally considered less toxic compared to synthetic organic antioxidants, they can potentially induce oxidative stress and lead to adverse health issues [20,21]. Moreover, the agglomeration of nanoparticles can significantly impact their practical application [22].

Herein, we report the discovery of the first soluble inorganic metal oxide cluster that exhibits synergistic antioxidant effects with thiols. Soluble metal oxide clusters can avoid issues such as toxicity and agglomeration associated with metal oxide nanoparticles. In our previous work, we developed a novel Sm-oxo/hydroxy cluster for applications as a catalyst in photoredox and thermal redox reactions, which prompted us to explore its potential as an antioxidant to scavenge oxidative radicals [23]. Our findings demonstrate that the in vitro antioxidant activities of a composition comprising Sm-oxo/hydroxy clusters and a cysteine derivative exhibited a robust inhibition of the oxidation process and displayed strong radical scavenging activity. Notably, due to its excellent photoredox activity, the antioxidant efficacy of the Sm-cluster/cysteine derivative was further enhanced under strong visible light conditions. While oxidation processes were accelerated under these conditions, the enhanced antioxidant activity under strong light suggests broader potential applications for this antioxidant strategy.

**Figure 1:**
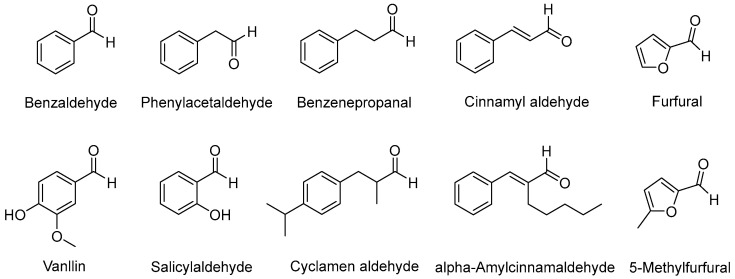
Some common aromatic aldehyde spices.

In this study, we specifically selected a series of aromatic aldehyde flavor compounds and DPPH (2,2-diphenyl-1-picrylhydrazine) to evaluate the antioxidant activity of the Sm-cluster/cysteine derivative composition. Aromatic aldehydes, such as benzaldehyde, benzyl aldehyde, vanillin, and cinnamaldehyde, naturally occur in many foods and food ingredients and are commonly used as flavorings and fragrances (Figure 1) [24]. These compounds are prone to free radical-initiated auto-oxidation when exposed to air, which can alter their sensory properties, produce toxic benzoic acid derivatives, cause food spoilage and color changes, and alterations in protein functionality, thereby reducing food quality [25]. Therefore, controlling their oxidative degradation is of significant concern in the food industry. Additionally, the DPPH assay is a well-established method for measuring radical scavenging activity [26], providing a reliable and quantifiable means to assess the antioxidant efficacy of the Sm-cluster/cysteine derivative composition. The combination of these tests allows for a comprehensive evaluation of the antioxidant potential and practical applications of our novel composition in improving food quality and stability.

## 2. Materials and Methods

### 2.1. Materials and Reagents

Sm metal (25 mesh) was purchased from Hebei Zhongyue Metal Materials Technology Co., Ltd. (Shijiazhuang, China) 1,2-Diiodoethane (98%) was purchased from Macklin. Benzaldehyde (98%), vanillin (98%), Methyl (tert-butoxycarbonyl)-L-cysteinate (*N*-Boc-L-Cysteine methyl ester, 90%), 6-hydroxy-2,5,7,8-tetramethylchroman-2-carboxylic acid (Trolox, 99%), Deuterated chloroform (CDCl_3_, 0.03 vol% tetramethylsilane (TMS), CAS no. 865–49-6, >99.8% D), Dimethyl sulfoxide-d6 (DMSO-*d*_6_, 0.03 vol% tetramethylsilane (TMS), CAS no. 2206-27-1, >99.8% D), and 1,1,2,2-Tetrachloroethane (99%) were purchased from Energy Chemical (Anqing, China). Cinnamaldehyde (98%) was purchased from Heowns (Tianjin, China). 1,1-Diphenyl-2-picrylhydrazyl radical 2,2-Diphenyl-1-(2,4,6-trinitrophenyl)hydrazyl (DPPH, 97%) was purchased from Aladdin (Shanghai, China). Deionized water was utilized in all experimental procedures, and oxygen was obtained from Jinghui Gas (Beijing, China). All solvents (analytical grade) are obtained from commercial sources and used as received, except as otherwise noted.

### 2.2. Instruments

Multifunctional microplate detector (BioTek Synergy HTX, BioTek Instruments, Inc., Winooski, VT, USA) and Bruker Avance NOE 500 (Bruker Corporation, Billerica, MA, USA). The SEM (scanning electron microscope) image of [Sm_6_O(OH)_8_(H_2_O)_24_]I_8_(H_2_O)_8_ cluster (Sm-cluster) was obtained on a Hitachi SU3500 (Hitachi High-Technologies Corporation, Tokyo, Japan). UV–vis diffuse reflectance spectroscopy was performed on a Hitachi U4150 (Hitachi High-Technologies Corporation, Tokyo, Japan). A 300 W Xe lamp (Zhongjiaojinyuan Co., Ltd., Beijing, China) was used as the artificial solar light source.

NMR data were measured with a Bruker Avance NOE 500 and manipulated directly from the spectrometer or via a networked PC with appropriate software. Reference values for residual solvent were taken as δ = 7.26 (CDCl_3_), δ = 2.50 (DMSO-*d*_6_) for ^1^H NMR. Multiplicities for coupled signals were designated using the following abbreviations: s = singlet, d = doublet, t = triplet, q = quartet, quin = quintet, br = broad signal, and are given in Hz.

### 2.3. Preparation of Sm-Cluster

Sm-cluster was prepared following the reported procedure in our previous work [23]. An excess amount of Sm metal (1.80 g, 12.0 mmol) was reacted with purified 1,2-diiodoethane (1.69 g, 6.00 mmol) in a 250 mL round-bottom flask. Carefully, argon gas was introduced into the flask for approximately 20 min to ensure an air-free atmosphere. Then, 60.0 mL of extra dry THF was added to the round-bottom flask containing Sm metal and 1,2-diiodoethane using syringes. The reaction mixture was stirred under argon atmosphere at room temperature. After 18 h, a dark blue SmI_2_ solution was formed. The reaction mixture was allowed to settle for 30 min to precipitate the unreacted Sm metal. Then, 24.0 mL of the SmI_2_ solution was transferred to a flask sealed with a rubber stopper and filled with Ar. Oxygen was carefully introduced to oxidize the SmI_2_ solution. After the solution turned yellow, deionized water (0.274 g, 15.2 mmol) was added under argon, and the mixture was stirred at room temperature for 5 h to obtain an orange-red solution. The solution was concentrated by rotary evaporation to remove most of the liquid. The remaining trace solvent was exposed overnight in a vacuum oil pump to yield a yellow solid Sm-cluster weighing 1.267 g. The structure of Sm-cluster was characterized through scanning electron microscopy (SEM) and ultraviolet–visible (UV–vis) diffuse reflectance spectroscopy.

### 2.4. Scanning Electron Microscope (SEM) of Synthesized Sm-Cluster

A crystal of the Sm-cluster was acquired using the layer diffusion method. A single crystal of the Sm-cluster was used to obtain the SEM image. Sm-cluster samples were mounted on aluminum stubs with sticky double-sided carbon tape and sputtered with a gold layer using an ion coater under vacuum. The samples were examined with a Hitachi SU3500 scanning electron microscope (Hitachi Ltd., Tokyo, Japan) operating at 30.0 keV.

### 2.5. Ultraviolet–Visible (UV–Vis) Diffuse Reflectance Spectroscopy of Sm-Cluster

In order to study the optical characteristics of synthesized Sm-cluster and verify its structure, the absorption spectrum was obtained on a Hitachi U4150 with an integrating sphere (Hitachi, Tokyo, Japan) in between a wavelength scan of 200–800 nm, using BaSO_4_ as the reflectance standard.

### 2.6. Evaluation of the Antioxidation Effect on Aromatic Aldehydes

To a quartz flask, aromatic aldehyde (0.200 mmol) and thiol (0.004–0.020 mmol, 2.00–10.0 mol%) were added, and the flask was sealed with rubber plug. Carefully, oxygen was introduced into the quartz flask for approximately 10 min to ensure an oxygen atmosphere. Then, Sm-cluster (1.3–6.6 mg, 0.10–1.0 mol%) dissolved in extra dry EtOAc was added to the system and then supplemented with EtOAc until a total volume of 6.0 mL. The mixture was stirred and illuminated with a 300 W Xe lamp under an oxygen atmosphere at room temperature for a period of 16 h. The mixture was concentrated under vacuum, and the recovery rate of aromatic aldehyde was determined through ^1^H NMR by an internal standard.

#### 2.6.1. ^1^H NMR Acquisition Method and Parameters

The concentrated mixture was dissolved in 0.5 mL of CDCl_3_ or DMSO-*d*_6_. The internal standard, 1,1,2,2-tetrachloroethane, was weighted and added to the solution. The solution was transferred into 5 mm NMR tubes for ^1^H NMR analysis. The one-dimensional ^1^H NMR spectra acquisition were recorded on a Bruker Avance NOE 500 MHz Digital FT-NMR Spectrometer (Peabody, MA, USA), equipped with a high-resolution, 5 mm wideband Z gradient probe. The ^1^H-NMR spectra were obtained using number of scans (NS) = 16, spectral width (SWH) of 10,000.0 Hz, size of fid (TD) = 32 K, relaxation delay = 1.0 s, and pulse width = 8.0 µs. And the spectra were processed using MestReNova 9.0.1 software (Santiago de Compostela, Spain, EU), where all spectra were Fourier transformed, zero filled to 64 K data points and the 0.2 Hz line broadening function was applied. Phase and baseline corrections were automatically performed.

#### 2.6.2. Calculation of the Recovery Rate by Internal Standard Using ^1^H NMR

First, 1,1,2,2-tetrachloroethane was used as the internal standard for yield calculations employing the ^1^H NMR internal standard approach. The procedure entailed the integration of ^1^H NMR signals corresponding to the target product and an internal standard of known quantity introduced into the reaction mixture. The ratio of the integrated areas of these signals furnished a quantitative assessment of product formation. Through comparison of this ratio to the known quantity of the internal standard, the yield of the desired product could be precisely ascertained. Recovery rates were determined via ^1^H NMR using the following equation:

(1) $$\mathrm{Yield}=\left(\frac{{\mathrm{Area}}_{\mathrm{reagent}}}{{\mathrm{Area}}_{\mathrm{internal}\;\mathrm{standard}}}\right)\left(\frac{n_{\mathrm{internal}\;\mathrm{standard}}}{n_{\mathrm{theoretical}\;\mathrm{reagent}}}\right)\times 100\%$$

Area_reagent_ represents the integration of the reagent peak, Area_internal standard_ represents the integration of the internal standard peak, n_internal standard_ represents the quantity of moles of the internal standard, n_theoretical reagent_ represents the theoretical quantity of moles of the reagent.

### 2.7. Evaluation of DPPH Free Radical Scavenging Activity

#### 2.7.1. General Method

The radical scavenging activity was evaluated through the DPPH radical scavenging assay, employing Trolox as the standard antioxidant compound. Experiments were conducted under dark conditions or xenon lamp irradiation. Utilizing the correlation between antioxidant activity and the standard, the obtained values were transformed from the equivalence of one specific antioxidant standard to another.

For the DPPH radical scavenging assay, sample **a** solution (0.1 mM *N*-Boc-L-Cysteine methyl ester and 0.2 mM Sm-cluster), sample **b** solution (0.1 mM *N*-Boc-L-Cysteine methyl ester), standard antioxidant **c** solution (0.1 mM Trolox), and the DPPH radical solution (50 mg/L) were all prepared with EtOAc. The sample or Trolox solutions with varying volumes (20, 40, 60, 80, and 100 μL) were added to a 2 mL DPPH radical solution, and a corresponding volume of EtOAc (980, 960, 940, 920, and 900 μL) was supplemented. The mixtures were then shaken vigorously and left to incubate for 30 min at room temperature under ambient indoor light or Xe lamp irradiation. For the blank group, the DPPH radical solution was replaced by EtOAc. For the control group, the sample or Trolox was replaced by EtOAc.

In a 96-well microplate, 200 µL of each mixture was added to each well. The absorbance was measured at 517 nm using a BioTek Synergy HTX microplate reader (BioTek Instruments, Inc., Winooski, VT, USA). The inhibition percentage of DPPH radical dot discoloration was calculated using the following Equation (2):

(2) $$\%\;\mathrm{Inhibition}=\frac{\left[1-\left(A_{sample}-A_{blank}\right)\right]}{A_{control}}\times 100\%$$

A_sample_ represents the absorbance of the samples, A_blank_ represents the absorbance of the blank (contained solvent instead of DPPH solution), A_control_ represents t the absorbance of the control (contained solvent instead of samples).

Trolox was used as the standard compound, and all the experiments were conducted in triplicate.

#### 2.7.2. Statistical Analysis

The results, presented as the means ± standard deviation (SD) of three replicate assays, were analyzed using SPSS 26.0 software (SPSS Inc., Chicago, IL, USA). Significant differences were determined by two-way analysis of variance (ANOVA) completed by Ducan’s test. Pearson’s correlation test was used to compare the different values of assays obtained in our extracts. Significant levels were defined at *p* < 0.05, *p* < 0.01, and *p* < 0.001. All these figures were performed by Origin 2024 (OriginLab Corporation, Northampton, MA, USA).

## 3. Results

### 3.1. Synthesis and Structural Characterization of Sm-Cluster

**Figure 2:**
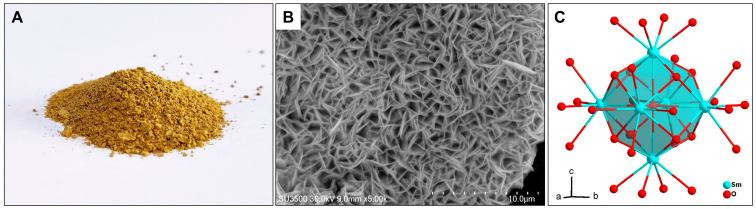
The Structure of Sm-cluster. (**A**) Image of Sm-cluster power; (**B**) SEM image of Sm-cluster single crystal; (**C**) Schematic of Sm-cluster Unit.

The Sm-cluster was synthesized using an auxiliary ligand-free oxidative hydrolysis method, and the product was obtained as a yellow-brown powder, as shown in Figure 2A. The SEM image (Figure 2B) of the Sm-cluster crystal revealed clear layered and hollow structures, with edge lengths of approximately 5–10 μm, consistent with previously reported data [23]. Further structural characterization was performed by UV–Vis diffuse reflectance spectroscopy (Figure S1). The results were also consistent with the reported data [23]. Those structural characterizations confirmed the successful synthesis of Sm-clusters with the expected structure (Figure 2C).

### 3.2. Evaluation of the Antioxidation Efficiency of Sm-Cluster/Cysteine Derivative

**Figure 3:**
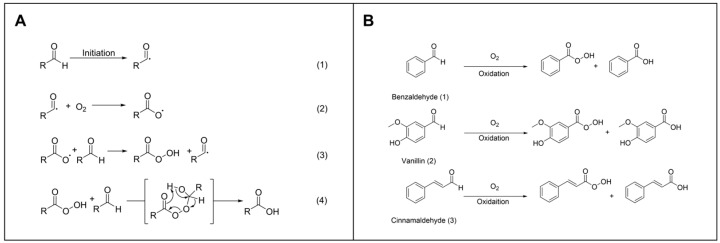
The autoxidation of aldehydes. (**A**) Mechanism of aldehydes autoxidation. (**B**) Reactions in the progress of selected aromatic aldehydes autooxidation.

To evaluate the antioxidation efficiency of the Sm-cluster/cysteine derivative, we selected three aromatic aldehydes—benzaldehyde, vanillin, and cinnamaldehyde—as model compounds due to their significance as flavor compounds. When aldehydes are exposed to oxygen, they can undergo autoxidation, which is a radical chain process (Figure 3A). This process typically begins with the formation of an aldehyde radical through hydrogen abstraction (Figure 3A, (1)), followed by the reaction with molecular oxygen to form a peroxy radical (Figure 3A, (2)). The peroxy radical can then react with another aldehyde molecule, generating a hydroperoxide and propagating the chain reaction (Figure 3A, (3)). This series of reactions can ultimately lead to the formation of peroxy acids or carboxylic acids, significantly affecting the stability and sensory properties of the aromatic aldehydes (Figure 3B).

The antioxidation efficiency of the Sm-cluster/cysteine derivative was rigorously evaluated under accelerated conditions. This involved simulating the autoxidation of aromatic aldehydes by exposing them to pure oxygen and/or irradiation with a 300 W Xe lamp for a duration of 16 h. The following four experimental groups were established: a blank control group, processing group **I** (with the addition of 1 mol% Sm-cluster), processing group **II** (with the addition of 2 mol% *N*-Boc-L-cysteine methyl ester), and processing group **III** (with the addition of 2 mol% *N*-Boc-L-cysteine methyl ester and 1 mol% Sm-cluster).

**Figure 4:**
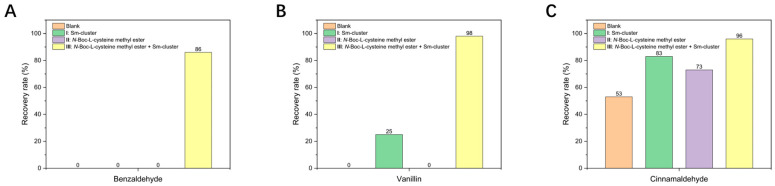
The recovery rates of three selected aromatic aldehydes with different processing groups. (**A**) Benzaldehyde. (**B**) Vanillin. (**C**) Cinnamaldehyde.

The recovery rates of aldehydes were determined by ^1^H NMR using 1,1,2,2-tetrachloroethane as the internal standard (Figure 4). The ^1^H NMR spectra of all the tested aromatic aldehydes listed below were used for the identification and quantification of the recovered aldehydes in the antioxidation tests (Figures S2–S4).

Benzaldehyde (**1**) [27]: ^1^H NMR (500 MHz, CDCl_3_) δ 10.00 (s, 1H), 7.86 (d, *J* = 8.0 Hz, 2H), 7.60 (t, *J* = 8.0 Hz, 1H), 7.51 (d, *J* = 8.0 Hz, 2H).

Vanillin (**2**) [28]: ^1^H NMR (500 MHz, DMSO-*d*_6_) δ 10.24 (s, 1H), 9.77 (s, 1H), 7.43–7.37 (m, 2H), 6.96 (d, *J* = 8.0 Hz, 1H), 3.84 (s, 3H).

Cinnamaldehyde (**3**) [29]: ^1^H NMR (500 MHz, CDCl_3_) δ 9.66 (d, *J* = 7.7 Hz, 1H), 7.54–7.50 (m, 2H), 7.42 (s, 1H), 7.41–7.36 (m, 3H), 6.73–6.62 (m, 1H).

**Figure 5:**
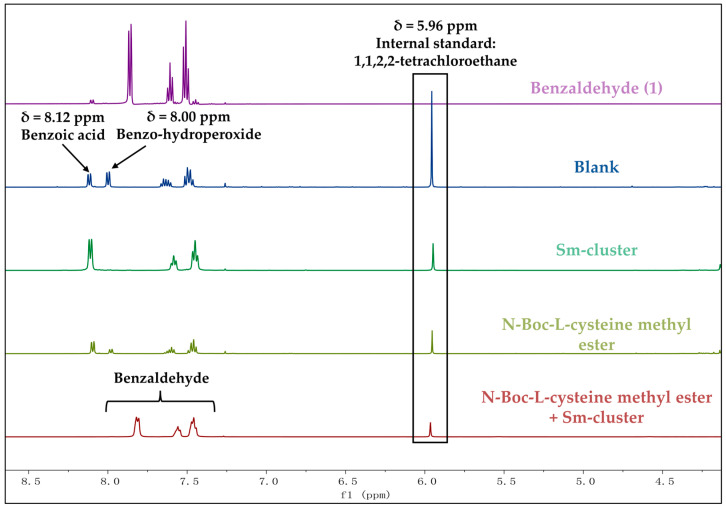
^1^H NMR spectra (CDCl_3_, 500 MHz) of experiments for benzaldehyde at room temperature (298 K).

Taking the benzaldehyde experimental group as an example, the ^1^H NMR spectra of pure benzaldehyde, the blank control group, and the processing groups are presented in Figure 5. The singlet observed at 5.96 ppm corresponds to the internal standard, 1,1,2,2-tetrachloroethane. The peak at 7.45 ppm is attributed to the meta-position hydrogen of the benzene ring in the aldehyde group, while the chemical shift at 7.55 ppm corresponds to the para-position hydrogen of the benzene ring. The peak at 7.80 ppm is assigned to the ortho-position hydrogen of the benzene ring. In the blank, **I**, and **II** groups, the peaks at 8.12 ppm and 8.00 ppm are assigned to the aromatic hydrogen of benzoic acid and benzohydroperoxide, respectively. The ^1^H NMR spectra clearly demonstrate that both benzoic acid and benzohydroperoxide were formed in a ratio of 1:1 in the blank group, with no benzaldehyde recovered. In groups **I** and **II**, although no benzaldehyde was recovered, benzoic acid was formed as the major oxidation product. In group **III**, no oxidation products were observed in the spectra.

The accurate recovery rates of benzaldehyde were calculated based on the peak area of the ortho-position hydrogen at 7.80 ppm relative to the peak area of the internal standard at 5.96 ppm. A similar method was employed to calculate the recovery rates of vanillin and cinnamaldehyde (Figures S5 and S6). The calculated recovery rates of the three aldehydes across the four experimental groups (Blank, **I**, **II**, and **III**) are illustrated in Figure 4.

According to the calculated recovery rates of aldehydes, the recovery rates in processing group **III** were significantly higher than those in the other groups. Specifically, even after exposure to pure oxygen under a 300 W Xe lamp for 16 h, the recovery rates of benzaldehyde, vanillin, and cinnamaldehyde were 86%, 98%, and 96%, respectively. In contrast, while cysteine derivatives are also commonly used antioxidants, the effectiveness was limited in this study. Only 0% of benzaldehyde and 25% of vanillin were recovered in processing group **II**. However, cinnamaldehyde, which is relatively more resistant to oxidation, showed an acceptable recovery rate of 83%. On the other hand, when the Sm-cluster was used alone (processing group **I**), it demonstrated minimal antioxidant effects. These findings clearly indicate the synergistic effect between the Sm-cluster and *N*-Boc-L-cysteine methyl ester, resulting in enhanced antioxidant efficacy. The combined use of Sm-cluster and cysteine derivative in processing group **III** significantly improved the stabilization and recovery rates of the aromatic aldehydes, even under harsh oxidative conditions. This synergistic interaction suggests a promising approach for improving the antioxidant properties of food preservatives.

### 3.3. Determination of Radical Scavenging Ability of Sm-Cluster/Cysteine Derivative

Free radical chain reactions are widely recognized as a common mechanism of lipid peroxidation (a major manifestation of food oxidation), including steps of initiation, reproduction, and chain termination [30]. The autoxidation of aldehydes is also a free radical chain reaction, following the same mechanism as free radical oxidation.

**Figure 6:**
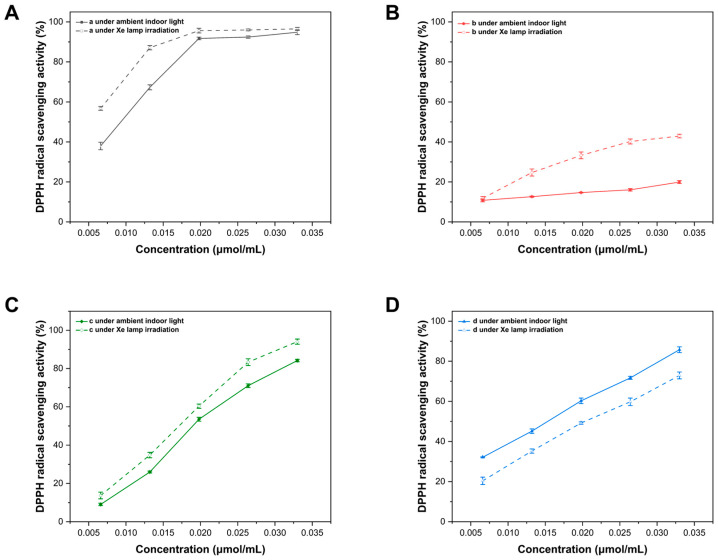
Determination of DPPH radical scavenging ability (%). (**A**) *N*-Boc-L-cysteine methyl ester + Sm-cluster. (**B**) *N*-Boc-L-cysteine methyl ester. (**C**) Sm-cluster. (**D**) Trolox.

Therefore, the ability of Sm-cluster/cysteine derivative to scavenge DPPH free radicals was measured to quantitatively represent its antioxidant capacity under ambient indoor light and 300 W Xenon lamp irradiation (Figure 6), where the Sm-cluster/*N*-Boc-L-cysteine methyl ester, *N*-Boc-L-cysteine methyl ester, Sm-cluster and Trolox were represented by **a**, **b**, **c** and **d**, respectively. Pearson’s test was used to find the correlation between concentration and antioxidant activity (Table 1). For both ambient indoor light and 300 W Xenon lamp irradiation, the DPPH free radical scavenging curves of Trolox exhibited better linearity (R^2^ = 0.998, 0.995, respectively), demonstrating the high reliability of this experiment, and their antioxidant activities were indexed in 50% inhibitory concentration (IC_50_) values (Table 2). A two-way ANOVA model was conducted using the IC_50_ value of different groups’ individual assays under two light conditions.

All samples quenched the DPPH free radicals in a dose-dependent manner. As the concentration of the samples increased, the DPPH scavenging activities also increased (Figure 6). Under ambient indoor light, the DPPH scavenging activities for the three samples, **a**, **b**, **c** and **d** were found to be in the following order: A_a_ > A_d_ > A_c_ > A_b_, where A_a_, A_b_, A_c_ and A_d_ were the DPPH scavenging activities for the sample **a**, **b**, **c** and **d**, respectively. Furthermore, the IC_50_ values of the samples were (8.49 ± 0.16) × 10^−3^, (1048.67 ± 43.25) × 10^−3^, (18.48 ± 0.26) × 10^−3^, and (14.41 ± 2.44) × 10^−3^ μmol/mL for **a**, **b**, **c**, and **d**, respectively. The DPPH radical scavenging ability of the *N*-Boc-L-cysteine methyl ester or Sm-cluster was much weaker than that of Trolox; however, the addition of Sm-cluster to the cysteine derivative resulted in a significant enhancement of DPPH free radical quenching activity. In addition, the lower IC_50_ values of **d** under Xe lamp irradiation ((5.70 ± 0.17) × 10^−3^ μmol/mL) showed that the DPPH free radical quenching activity of the Sm-clusters/cysteine derivative was further enhanced under Xe lamp irradiation, while the DPPH free radical quenching activity of positive control Trolox under Xe lamp irradiation (IC_50_ = (14.41 ± 2.44) × 10^−3^ μmol/mL) was lower than that under ambient indoor light (IC_50_ = (18.96 ± 0.37) × 10^−3^ μmol/mL).

All these results suggested that the addition of Sm-cluster was beneficial for elevating the antioxidant activity of *N*-Boc-L-cysteine methyl ester, and that the high illumination could promote the antioxidant activity of *N*-Boc-L-cysteine methyl ester, especially the combination of Sm-cluster and *N*-Boc-L-cysteine methyl ester. Given that Sm-cluster was known as a catalyst in photoredox and thermal redox reactions, it was suggested that the synergistic antioxidant effects of the cysteine derivative and Sm-cluster may also arise from Sm-cluster-catalyzed hydrogen atom transfer processes.

## 4. Conclusions

This study demonstrated the synergistic antioxidant effects of cysteine derivatives and Sm-cluster for food applications. The addition of soluble, low-toxic inorganic Sm-clusters significantly enhanced the antioxidant capacity of natural thiol compounds. The composition of Sm-oxo/hydroxy cluster and a cysteine derivative exhibited robust antioxidative effects, as evidenced by the inhibition of aromatic aldehyde oxidation and strong, dose-dependent DPPH radical scavenging activity. Notably, the antioxidant activity was further enhanced under strong visible light conditions. These findings suggest that the combination of inorganic clusters and thiol compounds presents a promising natural alternative to traditional synthetic antioxidants in the food industry.

## Figures and Tables

**Table 1:** Pearson‘s correlation analysis of DPPH scavenging activity of different groups. ** *p* < 0.01.

Groups	Minimal Light Exposure	Xenon Lamp Irradiation
a	0.897 **	0.820 **
b	0.973 **	0.968 **
c	0.993 **	0.992 **
d	0.998 **	0.995 **

**Table 2:** The IC_50_ values of DPPH scavenging activity of different groups. Different letters in the same column indicate significant differences (*p* < 0.01); ** *p* < 0.01.

Light Condition	Sample	IC_50_ (1 × 10^−3^ μmol/mL)
ambient indoor light	a	8.49 ± 0.16 ^b^
	b	1049 ± 43 ^a^
	c	18.48 ± 0.26 ^b^
	d	14.4 ± 2.4 ^b^
Xe lamp irradiation	a	5.70 ± 0.17 ^d^
	b	40.0 ± 1.2 ^a^
	c	15.35 ± 0.46 ^c^
	d	18.96 ± 0.37 ^b^
sample		**
light condition		**
sample × light condition		**

## Data Availability

The data presented in this study are contained within the article or in Supplementary Materials, or are available on request from the corresponding author Jie An.

## Supplementary Materials

The following supporting information can be downloaded at: https://www.mdpi.com/article/10.3390/antiox13080910/s1, Figure S1: UV–Vis diffuse reflectance spectroscopy of Sm-cluster; Figure S2: ^1^H NMR spectrum of benzaldehyde at room temperature (298 K); Figure S3: ^1^H NMR spectrum of vanillin at room temperature (298 K); Figure S4: ^1^H NMR spectrum of cinnamaldehyde at room temperature (298 K); Figure S5: ^1^H NMR spectra of experiments for vanillin at room temperature (298 K); Figure S6: ^1^H NMR spectra of experiments for cinnamaldehyde at room temperature (298 K).
