# Supplementary material for: Synergistic Antioxidant Effects of Cysteine Derivative and Sm-Cluster for Food Applications
Source: Antioxidants (Basel). 2024 Jul 28;13(8):910. doi: 10.3390/antiox13080910 (PMC11351330; doi:10.3390/antiox13080910)
Supplement: Supplementary file 1 [file antioxidants-13-00910-s001.zip › antioxidants-3114428-supplementary.pdf]

Supplementary Materials

Synergistic Antioxidant Effects of Cysteine Derivative and Sm-Cluster for Food Applications

Lingxia Chen [1], Lijun Wang [1], Lifu Ma [2] , Chao Wang [1], Xinshu Qin [1], Minlong Wang [1], Bing Fang [1, *], and Jie An [1,*]

[1] Department of Nutrition and Health, China Agricultural University, Beijing 100083, China; lxchen@cau.edu.cn (L.C.); lijunwang@cau.edu.cn (L.W.); b20233311213@cau.edu.cn (C.W.); b20233311203@cau.edu.cn (X.Q.); mlwang@cau.edu.cn (M.W.); bingfang@cau.edu.cn (B.F.)

[2] Tianjin Rianlon Corporation Research Institute Analytic Center，No.6, Huangshan Road, Modern Industrial Area, Tianjin Economic- Technological Development Area; malifu@rianlon.com (L.M.)

* Correspondence: jie_an@cau.edu.cn

Figure S1. UV–vis diffuse reflectance spectroscopy of Sm-cluster: Page 2

Figure S2. ^{1}H NMR spectrum of benzaldehyde at room temperature (298K): Page 3

Figure S3. ^{1}H NMR spectrum of vanillin at room temperature (298K): Page 3

Figure S4. ^{1}H NMR spectrum of cinnamaldehyde at room temperature (298K): Page 4

Figure S5. ^{1}H NMR spectra of experiments for vanillin at room temperature (298K): Page 5

Figure S6. ^{1}H NMR spectra of experiments for cinnamaldehyde at room temperature (298K): Page 5

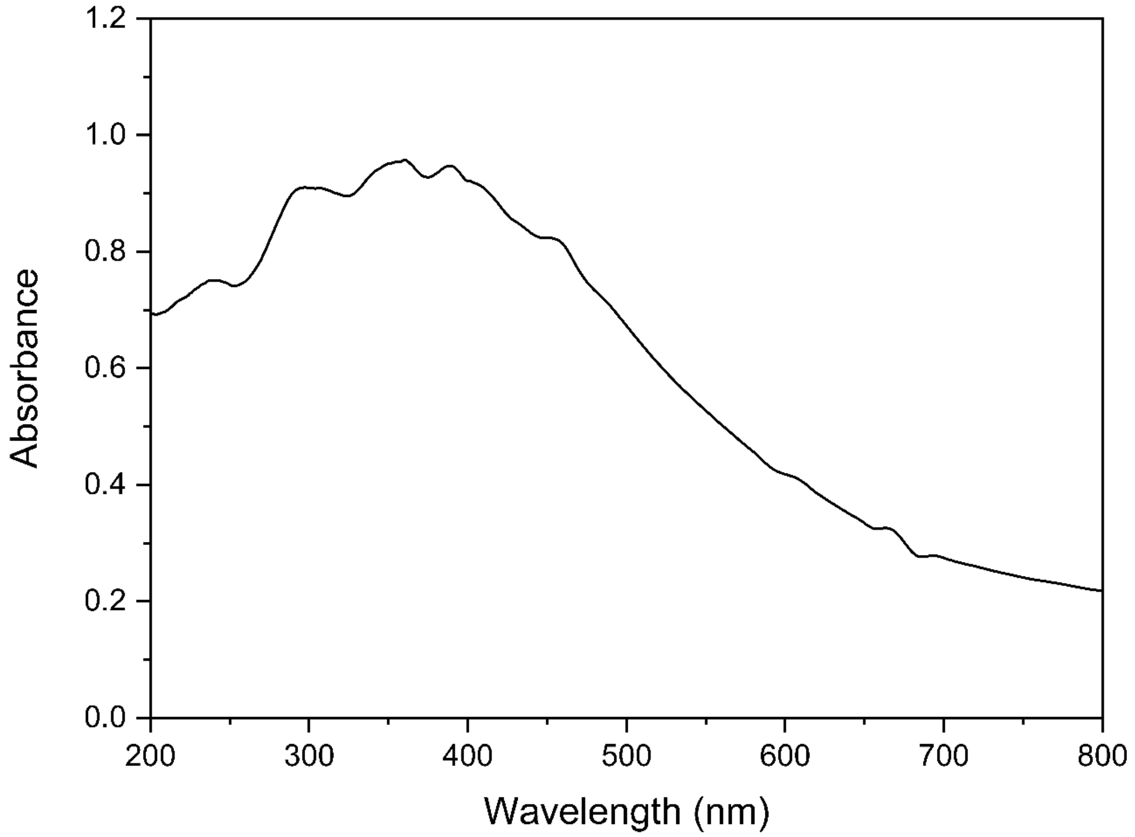

Figure S1. UV–vis diffuse reflectance spectroscopy of Sm-cluster.

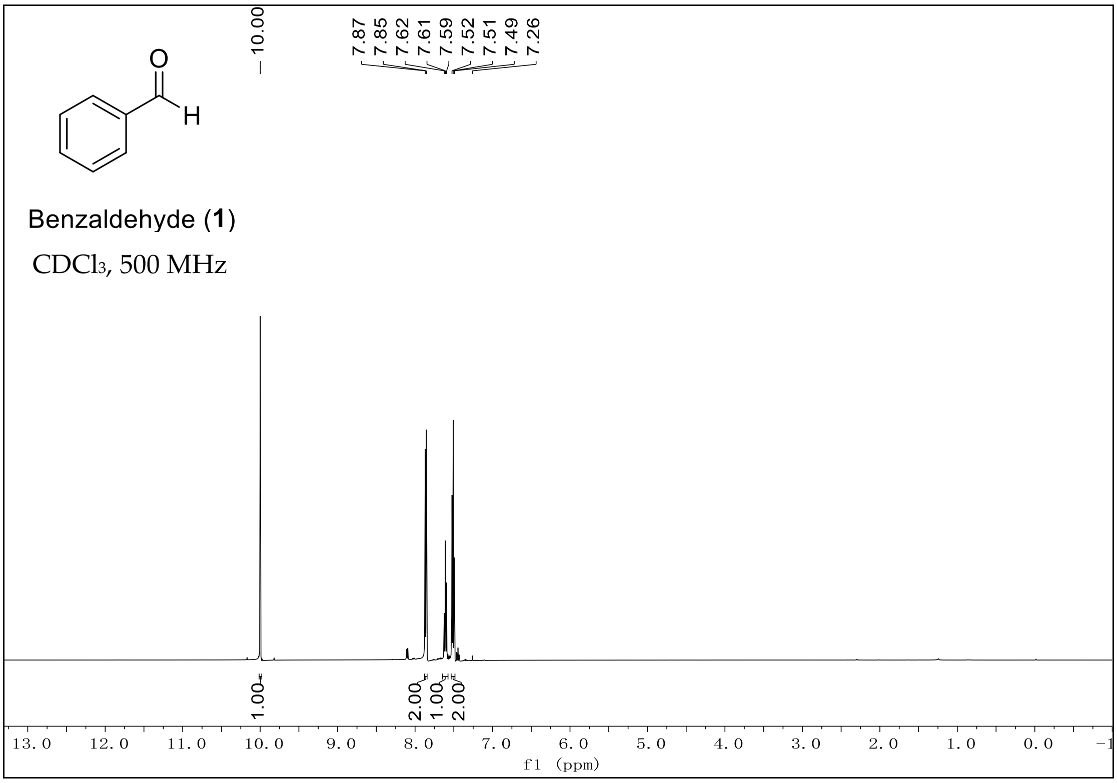

Figure S2. ^{1}H NMR spectrum of benzaldehyde at room temperature (298K).

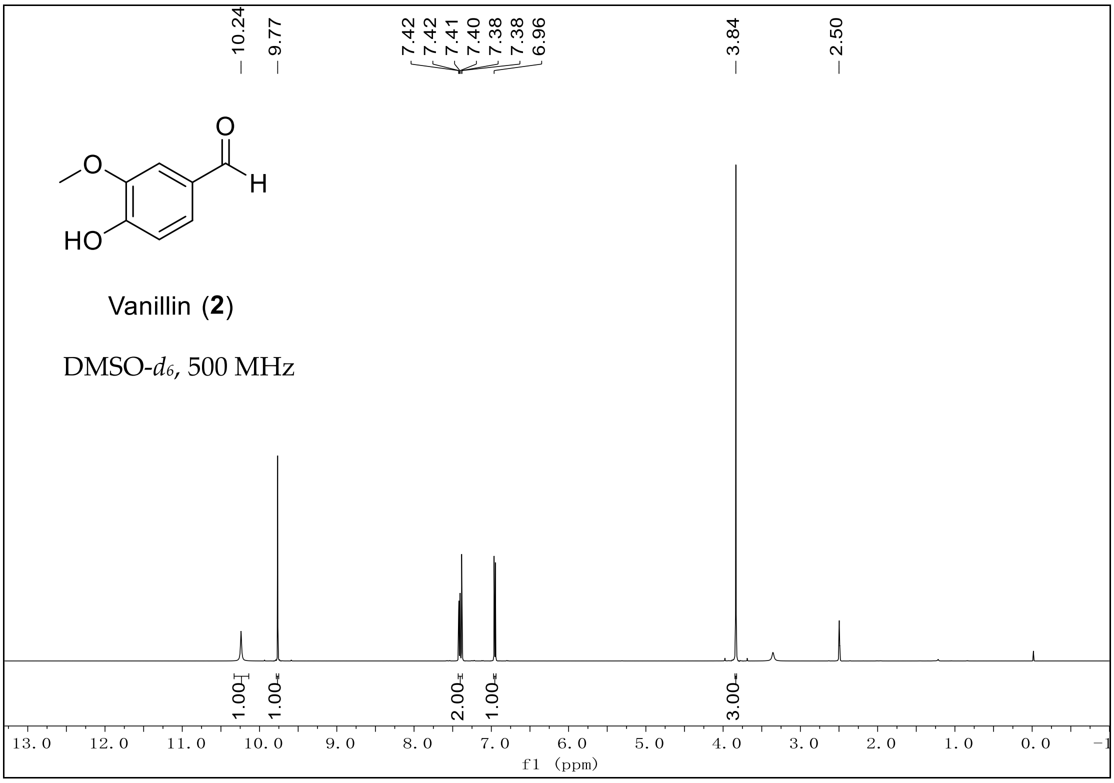

Figure S3. ^{1}H NMR spectrum of vanillin at room temperature (298K).

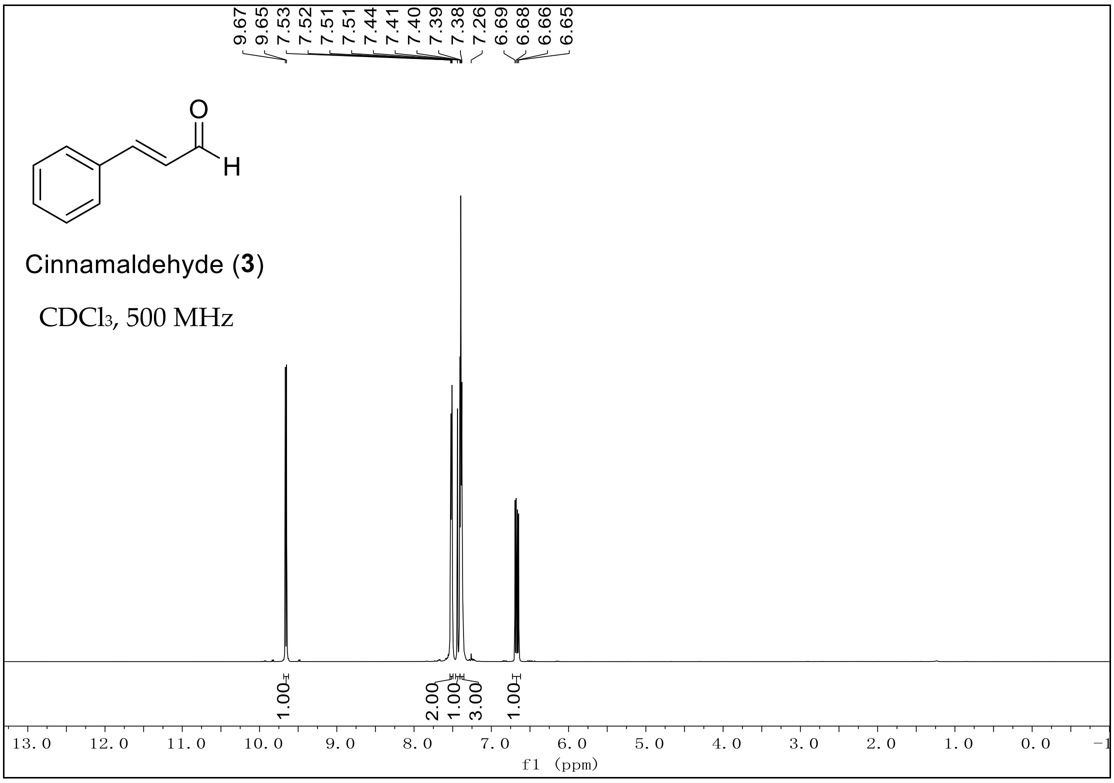

Figure S4. ^{1}H NMR spectrum of cinnamaldehyde at room temperature (298K).

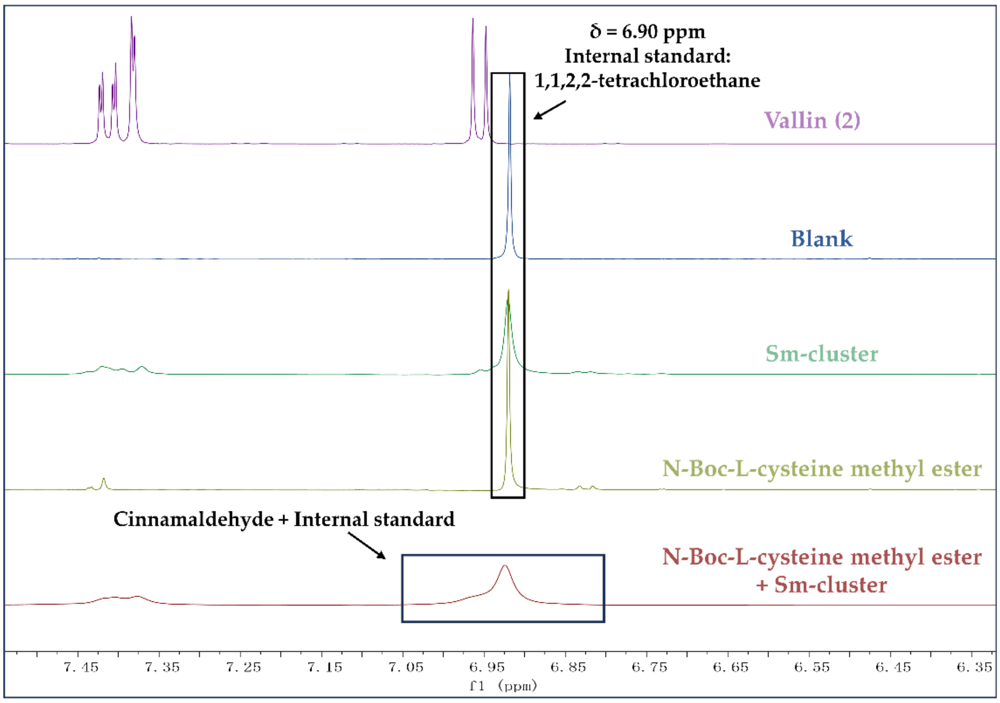

Figure S5. ^1H NMR spectra (DMso-d_6, 500 MHz) of experiments for vanillin at room temperature (298K).

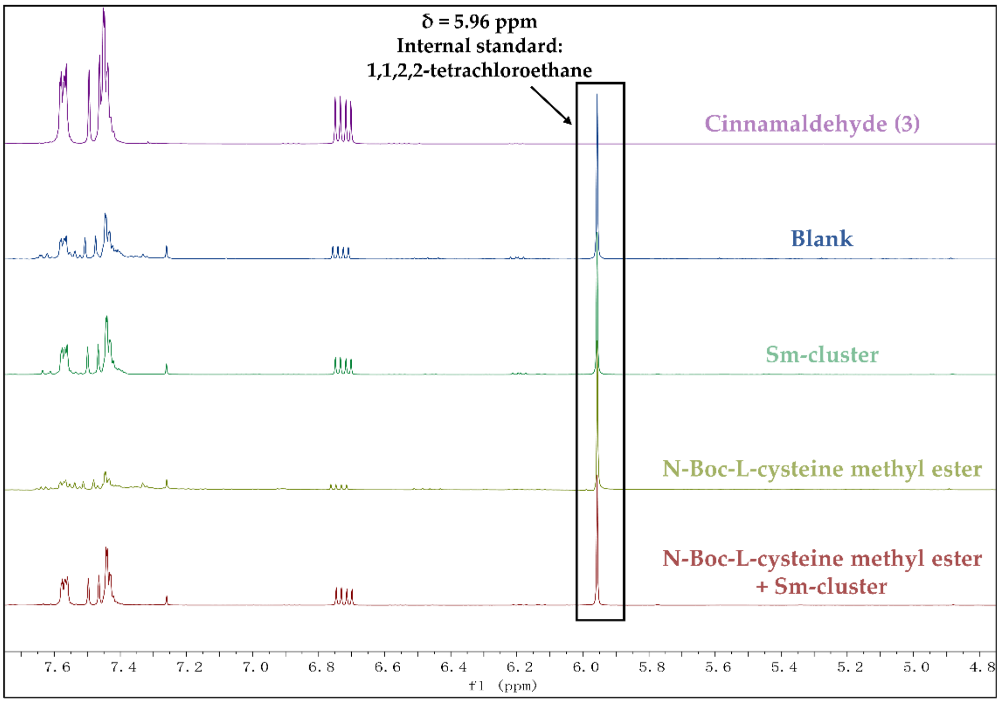

Figure S6. ^1H NMR spectra ($CDCl_3$, 500 MHz) of experiments for cinnamaldehyde at room temperature (298K).
